# NLRP3 exacerbates EAE severity through ROS-dependent NET formation in the mouse brain

**DOI:** 10.1186/s12964-023-01447-z

**Published:** 2024-02-02

**Authors:** Da Jeong Byun, Jaeho Lee, Kyungryung Ko, Young-Min Hyun

**Affiliations:** Department of Anatomy and Brain Korea 21 PLUS Project for Medical Science, Yonsei University College of Medicine, Seoul, Republic of Korea

**Keywords:** EAE, Neutrophil, NET, Inflammation, NLRP3 inflammasome

## Abstract

**Background:**

Neutrophil extracellular trap (NET) has been implicated in the pathology of multiple sclerosis (MS) and experimental autoimmune encephalomyelitis (EAE). However, the specific contributions of NLRP3, a NET-associated molecule, to EAE pathogenesis and its regulatory role in NET formation remain unknown.

**Methods:**

To investigate the detrimental effect of NETs supported by NLRP3 in MS pathogenesis, we induced EAE in WT and NLRP3 KO mice and monitored the disease severity. At the peak of the disease, NET formation was assessed by flow cytometry, immunoblotting, and immunofluorescence staining. To further identify the propensity of infiltrated neutrophils, NET-related chemokine receptors, degranulation, ROS production, and PAD4 expression levels were evaluated by flow cytometry. In some experiments, mice were injected with DNase-1 to eliminate the formed NETs.

**Results:**

Our data revealed that neutrophils significantly infiltrate the brain and spinal cord and form NETs during EAE pathogenesis. NLRP3 significantly elevates NET formation, primarily in the brain. NLRP3 also modulated the phenotypes of brain-infiltrated and circulating neutrophils, augmenting CXCR2 and CXCR4 expression, thereby potentially enhancing NET formation. NLRP3 facilitates NET formation in a ROS-dependent and PAD4-independent manner in brain-infiltrated neutrophils. Finally, NLRP3-supported NET formation exacerbates disease severity, triggering Th1 and Th17 cells recruitment.

**Conclusions:**

Collectively, our findings suggest that NLRP3-supported NETs may be an etiological factor in EAE pathogenesis, primarily in the brain. This study provides evidence that targeting NLRP3 could be a potential therapeutic strategy for MS, specifically by attenuating NET formation.

**Supplementary Information:**

The online version contains supplementary material available at 10.1186/s12964-023-01447-z.

## Background

Multiple sclerosis (MS), an autoimmune disease, significantly affects the central nervous system (CNS), leading to neurodegeneration [1–3]. This disease is characterized by sporadic demyelination of the CNS and neurological dysfunction, resulting in symptoms such as limb weakness, ataxia, and impaired cognition [4]. Despite affecting nearly two million people worldwide, MS lacks a definitive cure due to its intricate pathology [5]. Experimental autoimmune encephalomyelitis (EAE) serves as the primary model for studying MS due to its significant clinical and scientific parallels [6, 7]. Most EAE model-based MS studies have focused on autoreactive myelin-specific T-cells that trigger the inflammatory cascade and demyelination [3, 8]. However, recent evidence notes the involvement of other innate immune cells, including neutrophils, in MS progression [9, 10]. Particularly, neutrophils have been reported to infiltrate to the CNS and could exert a deleterious effect on the pathogenesis of MS, as supported by the finding that neutrophil depletion mitigates EAE progression [1, 9]. Hereby, our attention was directed towards the peak of the disease to explore not only the dynamics of neutrophils in the pathogenesis of EAE but also the interaction between NET formation and adaptive T cells in this investigation. Furthermore, patients with MS exhibit hyperactivated phenotypes of neutrophils, characterized by amplified generation of reactive oxygen species (ROS) and degranulation, as well as higher levels of neutrophil extracellular trap (NET) in the serum, compared to healthy individuals [1]. Therefore, elucidating this complicated pathology through laboratory research holds significant promise for guiding clinical approaches to benefit MS patients, with a particular focus on identifying the effects of neutrophils, while providing valuable insights into its pathogenesis.

Among the versatile functions of neutrophils, we focused on the NET as an etiological factor, which has been implicated in exacerbation of several diseases, contrary to its original beneficial function of inhibiting the spread of pathogens throughout the body [11, 12]. Upon stimulation, neutrophils process intracellular signals such as ROS, myeloperoxidase (MPO), neutrophil elastase (NE), and protein arginine deiminase 4 (PAD4), leading to chromatin decondensation and histone complex citrullination [13, 14]. Released NETs possess sticky properties that enable them to accumulate in inflamed tissues and cause tissue toxicity while stimulating other immune cells and cytokines [13, 15]. Previous studies have reported the presence of NET components in the blood of patients with a relapsing–remitting disease course, which has also been observed in several other autoimmune diseases [16, 17]. Therefore, investigating NET regulation may offer new therapeutic avenues for MS.

Herein, we hypothesized the role of the NLR family pyrin domain containing 3 (NLRP3) in NET formation upon EAE induction. As a pivotal intracellular molecule in neutrophils responsible for signaling immune responses, NLRP3 senses various stimuli through pathogen-associated molecular patterns (PAMPs) and damage-associated molecular patterns (DAMPs) [18, 19]. Considering that neutrophils initiate NET formation after recognizing these signals, it is plausible that NLRP3 is also associated with NET formation [20]. Supporting this assumption, NLRP3 inflammasome dysregulation has been linked to several pathological conditions characterized by excessive NET formation, including familial Mediterranean fever and Muckle–Wells Syndrome [21–23]. In addition, we have previously demonstrated that the inhibition of NLRP3 ameliorates neuroinflammation elicited by excessive NET formation in the lipopolysaccharide (LPS)-induced inflamed brain [24]. Furthermore, several studies have shown that NLRP3 promotes EAE progression, as evidenced by the alleviation in EAE severity in the absence of NLRP3 [25, 26]. However, the relationship between NET formation facilitated by NLRP3 and EAE progression remains poorly understood.

In other words, while NETs, NLRP3, and MS are individually associated, the therapeutic potential of targeting NLRP3 to control NET formation in MS remains unexplored. Hence, this study aimed to investigate the regulatory role of NLRP3 in NET formation in the EAE-induced CNS and assess its impact on EAE progression.

## Materials and methods

### Mice

C57BL/6 mice were purchased from Orient Bio (Daejeon, Korea). NLRP3-deficient (NLRP3 KO) mice were provided by Professor Je-Wook Yu of Yonsei University, Seoul, Republic of Korea [27]. Mice were bred and housed in a specific pathogen-free animal facility at the Avision Biomedical Research Center, Yonsei University College of Medicine. All experiments were performed in accordance with the approved guidelines of the Institutional Animal Care and Use Committee of Yonsei University College of Medicine (IACUC No. 2022–0116).

### Antibodies

Anti-mouse Ly6G (IA8; 127608; BioLegend, CA, USA), anti-mouse CD11b (M1/70; 101212; BioLegend), anti-mouse CD63 (NVG-2; 143920; BioLegend), anti-mouse CXCR2 (SA044G4; 149315; BioLegend), anti-mouse CXCR4 (L276F12; 146511; BioLegend), and anti-mouse CD4 (GK1.5; 100406; BioLegend) antibodies were used to stain the surface molecules of samples. For intracellular staining, anti-mouse IFN-γ (XMG1.2; 505809; BioLegend) and anti-mouse IL-17A (TC11-18H10.1; 506903; BioLegend) antibodies were used. For NET components staining, anti-mouse MPO (ab90812; Abcam, MA, USA), anti-mouse CitH3 (ab5103; Abcam), anti-mouse PAD4 (ab96758; Abcam), anti-mouse NE (ab38672; Abcam), anti-mouse β-actin (8457S; Cell Signaling Technology, MA, USA), Alexa Fluor 488 goat anti-rabbit IgG (ab150077; Abcam), Alexa Fluor 555 goat anti-rabbit IgG (ab150078; Abcam), and Alexa Fluor 647 donkey anti-rabbit IgG (101,231; BioLegend) antibodies were used.

### EAE Induction

The MOG_35-55_/CFA Emulsion PTX kit (Hooke Labs, Lawrence, MA, USA) was used to induce EAE, according to the manufacturer’s protocol. Ten- to twelve-week-old female C57BL/6 or NLRP3 KO mice were immunized subcutaneously at two sites on their back with MOG_35-55_ emulsified in complete Freund’s adjuvant (CFA). Subsequently, pertussis toxin (PTX) was injected intraperitoneally twice, 2 and 24 h after the prior immunization. EAE-induced mice were then randomly allocated to each treatment group. The EAE clinical scores were given daily in a blinded fashion by three independent observers as follows; 0, no obvious signs of disease; 0.5, partially limp tail; 1, complete limp tail; 1.5, limp tail and waddling gait; 2, limp tail and complete paralysis of one hind limb; 2.5, limp tail, complete paralysis of one hind limb, and partial paralysis of the other hind limb; 3, limp tail and complete paralysis of both hind limbs; 3.5, limp tail, complete paralysis of both hind limbs, and ascending paralysis; 4, paralysis of trunk; 4.5, moribund; and 5, dead [28, 29].

### Single-cell dissociation from the brain and spinal cord

To obtain leukocytes from the brain and spinal cord, tissues from each group were homogenized after transcardial perfusion with cold 1 × phosphate-buffered saline (PBS). Isolated spinal cords were treated with 1 mg/mL of DNase 1 (10 104 159 001; Sigma‐Aldrich) and 1 mg/mL of Collagenase D (11 088 866 001; Sigma‐Aldrich) at 37 °C, followed by incubation at 80 RPM on a shaker for 40 min. Subsequent to enzyme digestion, 500 mM ethylenediaminetetraacetic acid (EDTA) was treated. Homogenized brains and spinal cords were sieved through a 70-μm pore nylon mesh (SPL, Gyounggi-do, South Korea). To dissociate the brain and spinal cord cell population, stock isotonic Percoll (SIP) was prepared by adding nine parts of pure Percoll (Percoll Plus, GE Healthcare, Uppsala, Sweden) to one part of 10 × Hanks’ balanced salt solution (HBSS). The acquired pellet was resuspended in 10 mL 30% SIP and slowly overlaid on top of 70% SIP. The gradient was centrifuged at 500 × g for 30 min at 20 °C. After centrifugation, the myelin debris were removed, and a 70%–30% interface was taken. The cells were then washed twice with 1 × HBSS [30].

### Flow cytometry

Prior to antibody staining, cell suspensions were incubated with purified anti-mouse CD16/CD32 (93, BioLegend, San Diego, CA, USA) to prevent non-specific immunoglobulin binding to Fc receptors. Suspended cells were stained with Zombie Aqua Fixable Viability Kit (BioLegend) to exclude dead cells, incubating at 20℃ for 10 min in advance of antibody staining. To detect cell surface molecules and released NETs, cell pellets were stained with each antibody in PBS containing 2% FBS and 2 mM EDTA for 20 min at 4 °C. Particularly, extracellular CitH3 and PAD4 expressions were detected by performing separate primary (1:500) and secondary antibody (1:500) staining for 20 min at 4 °C [31]. To detect ROS production, the cells were incubated with 20 µM DCFDA (ab113851; Abcam), a general oxidative stress indicator, for 30 min at 37℃ [18, 32, 33]. The cells were incubated with a Cell Stimulation Cocktail (plus a Protein Transport Inhibitor) (eBioscience, San Diego, CA, USA) for intracellular staining. The cells were stained with anti-mouse CD4 and permeabilized using a Foxp3/Transcription Factor Fixation/Permeabilization Kit (Thermo Fisher, CA, USA) following the manufacturer’s instructions. Intracellular cytokines were stained with permeabilization buffer (Thermo Fisher, CA, USA).

### RNA extraction and quantitative real-time PCR (qRT-PCR)

Total RNA was prepared using TRIzol Reagent (Invitrogen, Carlsbad, CA, USA) according to the manufacturer’s instructions. Next, 2000 ng of total RNA was reverse transcribed into cDNA using the AccuPower CycleScript RT Premix (Bioneer, Daejeon, Korea). The mRNA expression level of each gene was measured using the SYBR-Green System and QuantStudio3 system (Applied Biosystems) according to the standard protocol. All data were normalized to *Tbp* expression [34].

### In vivo NET Inhibition

To eliminate the formed NETs, the mice were simultaneously injected with 10 µg i.v. and 50 µg i.p. human recombinant DNase-1 (enz-319-10000 IU; ProSpec, Israel) [35, 36], from day 7 post-immunization, and then 50 µg i.p. every 24 h until sacrifice at the peak EAE stage. As a control, mice were injected with vehicle (8.77 mg/mL sodium chloride and 0.15 mg/mL calcium chloride).

### Immunofluorescence

The mice were anesthetized and transcardially perfused with cold PBS. We harvested entire brain except olfactory bulb and thoracic to lumbar part of spinal cord. The obtained brains and spinal cords were fixed in 10% formalin solution (Sigma-Aldrich, Germany) at 4℃ overnight. The fixed tissues were placed in a 30% sucrose solution until the tissues settled to the bottom at 4℃. The tissues were then embedded in an optimal cutting temperature compound (OCT, Leica Biosystems, IL, USA) and cut into 10-µm thick sections. Sectioned tissues were washed thrice in PBS to remove residual OCT compounds. The tissues were permeabilized and non-specific binding was blocked using 1% bovine serum albumin (BSA), 2 mM EDTA, and 0.5% Triton X-100 in PBS for 1 h at 25℃.The tissues were incubated overnight at 4℃ with primary antibodies diluted in the same media to detect NET. The samples were rinsed thrice with PBS and then incubated with secondary antibodies: Alexa Fluor 488 goat anti-rabbit IgG (2 μg/mL) and Alexa Fluor 555 goat anti-rabbit IgG (2 μg/mL) for 2 h at 25℃ [24].

### Immunoblotting Analysis

Total protein was acquired from the brains and spinal cords, following a previously described method. The mice were perfused before sacrifice and the tissues were homogenized. The PRO-PREP protein extraction solution (Intron Biotechnology, Korea) was used for whole-tissue protein extraction. To measure NET formation from the proteins, the PVDF immunoblotting membrane was incubated with primary antibodies overnight at 4 °C. After rinsing the residual primary antibodies, the target proteins were incubated with an HRP-conjugated secondary antibody for 1 h at 25 °C. The membrane was developed using Clarity Max Western ECL substrate (Bio-Rad, Hercules, CA, USA) and the target bands were captured using an ImageQuant LAS 4000 mini (Fujifilm, Tokyo, Japan). Data were analyzed using the ImageJ software (NIH, USA) [12].

### Luxol fast blue staining

To evaluate demyelination in the mouse brain and spinal cord, fixed tissues were stained using the Luxol Fast Blue Stain Kit (Abcam), as described in the manufacturer’s protocol. Mice were sacrificed and assessed at the peak EAE stage, approximately 17–19 days post immunization. The sections were incubated with 0.1% Luxol fast blue solution at 24℃ overnight and differentiated in 0.05% lithium carbonate solution at 24℃. When differentiation was completed, the sections were counterstained with 0.1% cresyl echt violet solution for 3 min at 24℃. Analysis of demyelination was conducted in both the brain corpus callosum region and the thoracic to lumbar region of spinal cord. The demyelinated regions in the white matter were quantified using ImageJ (NIH, USA) [37, 38].

### Statistical analysis

Statistical analysis was performed with Prism v7.0 (GraphPad). For comparing with two or more samples, student’s t-test or one-way ANOVA was conducted. Grouped samples were analyzed with two-way ANOVA and Tukey’s multiple comparison post-test was also performed. Data are presented as mean with S.E.M. *P* values < 0.05 were considered significant (*P < 0.05, **P < 0.01, ***P < 0.001, ****P < 0.0001).

## Results

### Neutrophils significantly infiltrated and generated NETs in EAE-induced CNS

**Fig. 1:**
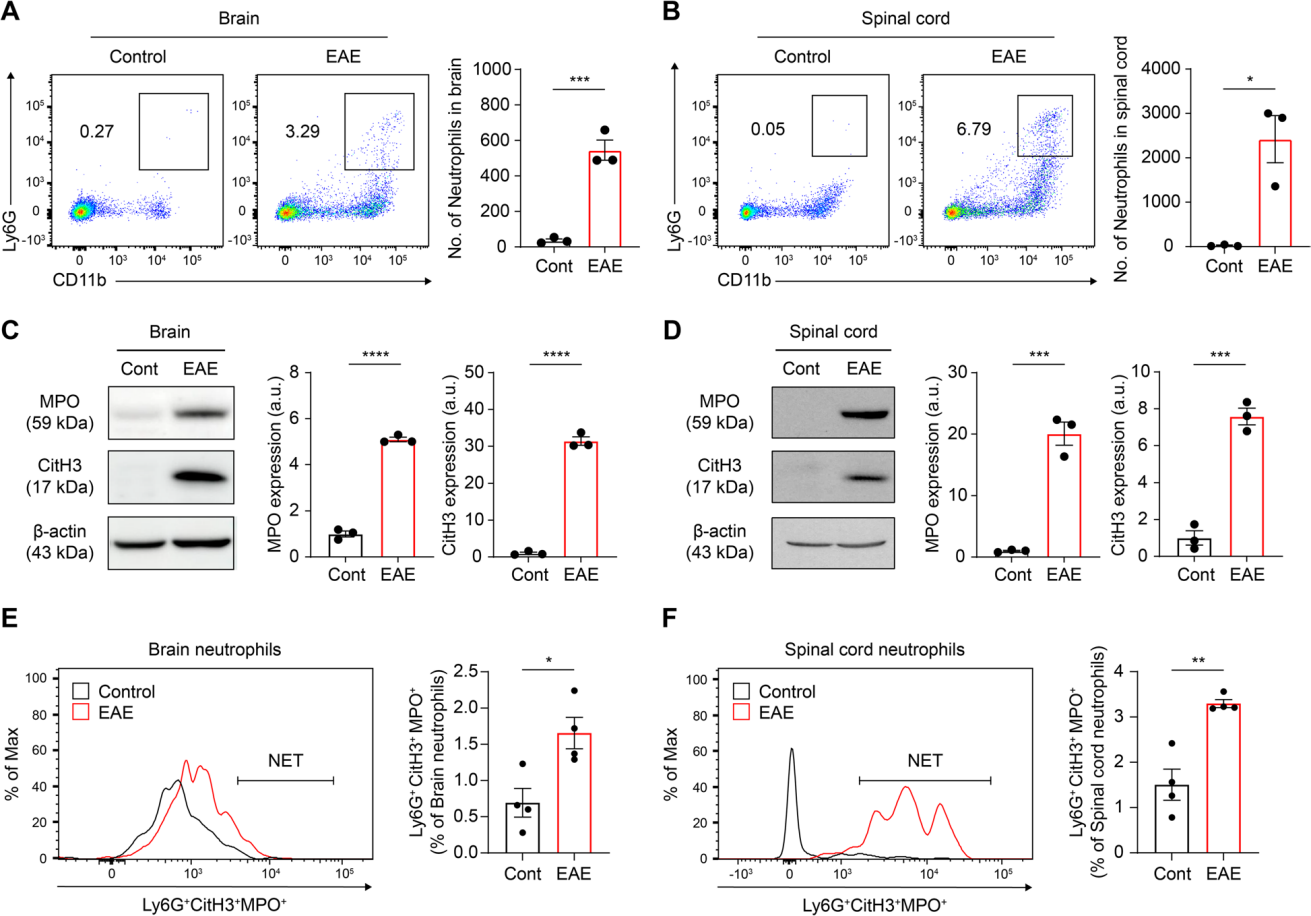
Neutrophils and their extracellular traps were substantially observed in the CNS during EAE pathogenesis. **A**–**F** Identification of neutrophil infiltration and NET formation in the EAE-induced CNS, as compared to control. Representative scatter plots and the absolute number of infiltrated neutrophils in the (**A**) brain and (**B**) spinal cord. Immunoblotting and densitometric analysis for MPO, CitH3, and β-actin in the (**C**) brain and (**D**) spinal cord. For quantification, the target expressions were normalized using β-actin. Representative histogram of the percentage of NET formation (Ly6G^+^CitH3^+^MPO.^+^) and graph in the (**E**) brain and (**F**) spinal cord. Cont, control. Data are obtained from at least triplicated repeat and presented as mean ± SEM. *P < 0.05, **P < 0.01, ***P < 0.001, ****P < 0.0001

First, we compared the numbers of infiltrated neutrophils in the CNS of EAE-induced and healthy control mice to confirm the key role of neutrophils in disease pathogenesis. Infiltrated neutrophils were identified as CD11b- and Ly6G-positive subsets within CNS myeloid cells through flow cytometry. Although neutrophils are prominent first-line responders and short-lived myeloid cells, we observed a substantial number of neutrophil infiltrations in the brain and spinal cord between days 17–19 post-immunization, which corresponded to the disease peak (Fig. 1A and B). Recruited neutrophils have various effects on the pathogenesis of MS; however, we focused on NET formation as a possible deleterious effect [13]. Emitted NET accumulates in lesions, which has been reported as an etiological factor, particularly in autoimmune diseases, such as experimental lupus and rheumatoid arthritis [39, 40]. Moreover, according to clinical data, a subset of patients with relapsing–remitting disease course exhibit circulating NETs in their sera [16]. We directly evaluated the level of NET formation in the EAE-induced CNS. Hence, we employed citrullinated histone H3 (CitH3) as a specific hallmark of NETs, which is derived during the NET formation process in which chromatin decondenses and arginine is converted into citrulline within the histone complex [14, 41]. Furthermore, we used MPO, an intracellular molecule that exists in myeloid cells and is leaked as a component of NET matrices, as a NET indicator [42]. We assessed NET-indicators expression in the CNS at protein level using immunoblotting. Notably, MPO and CitH3 showed apparent expression in the EAE-induced group in both the brain and spinal cord at disease peak (Fig. 1C and D). Moreover, the proportion of NET formation, which was identified using MPO and CitH3 double-positive neutrophils, was assessed [24, 31]. Specifically, to define NET-generating population, we initially gated Ly6G-positive neutrophils in the inflamed brain leukocytes, followed by gating CitH3. Subsequently, among the CitH3-positive population, we gated MPO-positive, representing the co-localized population of Ly6G, CitH3, and MPO, in accordance with a previously reported [31] (Supplementary Fig. 1). These results were consistent with the protein level examination, indicating that the EAE progression was accompanied by NET formation within the CNS (Fig. 1E and F). Particularly, our findings suggest that EAE, a type of autoreactive chronic neuroinflammation, causes substantial neutrophil infiltration and the subsequent accumulation of NETs in the CNS. This emphasizes the crucial role of neutrophils and NETs in disease pathology and reinforces the requirement for further investigation of neutrophil-centric interventions in the management of EAE.

### NLRP3 deficiency alleviates EAE severity, but is extraneous to neutrophil infiltration in the CNS at disease peak

**Fig. 2:**
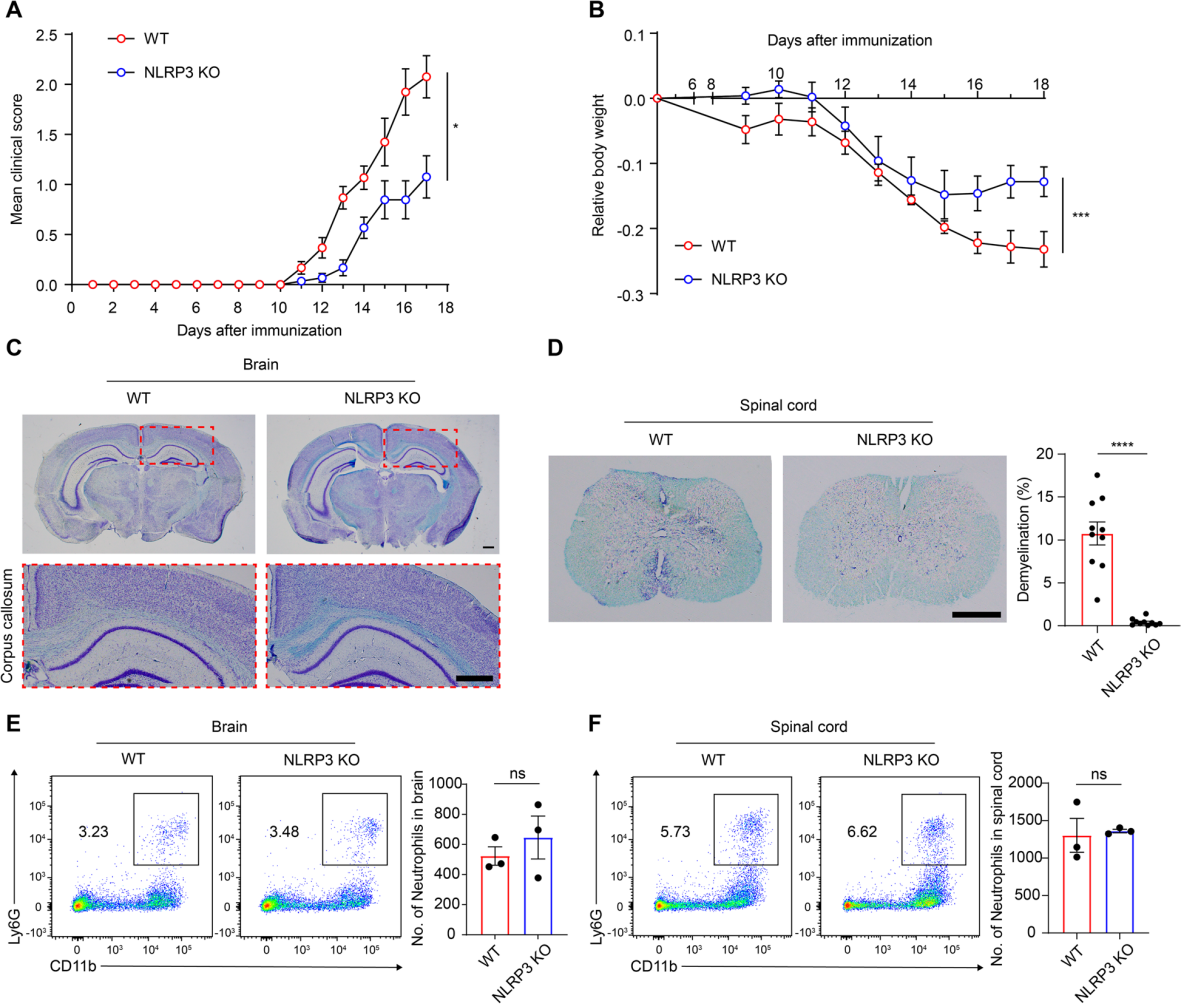
NLRP3 exacerbates EAE severity, but is irrelevant to neutrophil infiltration into the CNS during EAE peak. **A**–**F** Comparison of EAE severity and CNS-infiltrated neutrophil number between WT and NLRP3 KO mice. **A** Clinical scores of EAE from both groups. The combined results of three independent experiments are shown. **B** Relative body weight in both groups following EAE induction. Both groups (**A** and **B**) were conducted with 15 mice each. (C and D) A comparison of brain demyelination between both groups at the peak of the disease, stained with luxol fast blue staining. **C** Upper, representative images of brain sections. Lower, the demyelinated area in the corpus callosum of WT and NLRP3 KO brains. **D** Left, representative images of, spinal cord demyelination in both groups at the disease peak. Scale bar = 500 μm. Right, the analysis of demyelination degree in both groups through thoracic to lumbar part of spinal cord. Representative scatter plots and the absolute number of infiltrated neutrophils in the **E** brain and **F** spinal cord of both groups at peak disease. ns, non-significant. Data are driven from at least triple replicates and presented as mean ± SEM. *P < 0.05, ***P < 0.001, ****P < 0.0001

Next, we explored the potential link between EAE pathogenesis and NLRP3 inflammasome, a molecule closely associated with NET formation. Our investigation confirmed that the absence of NLRP3 effectively mitigated the severity of EAE, as previously reported [26, 43]. This was evidenced by a significant reduction in EAE symptoms, prevention of weight loss, and a decrease in demyelination (Fig. 2A–D). Given this attenuation of disease severity, we sought to elucidate the relationship between NLRP3 and neutrophils in the context of MS. Considering the previously observed increase in neutrophil counts in the CNS, we evaluated the effect of NLRP3 deficiency on neutrophil infiltration during disease progression. Despite reduced disease severity due to NLRP3 deficiency, the numbers of infiltrated neutrophils in WT and NLRP3 KO mice were indistinguishable in both the brain and spinal cord at the disease peak (Fig. 2E and F). These findings led us to speculate that NLRP3 could affect neutrophil functions, such as NET formation, thereby aggravating the severity of EAE. Our hypothesis is further supported by previous studies reporting that accumulated NETs can exacerbate autoimmune diseases and that NLRP3 can enhance NET formation in septic neuroinflammation [17, 24, 39].

### NLRP3 deficiency mainly alleviated NET formation in the brain, but not the spinal cord, during EAE progression

**Fig. 3:**
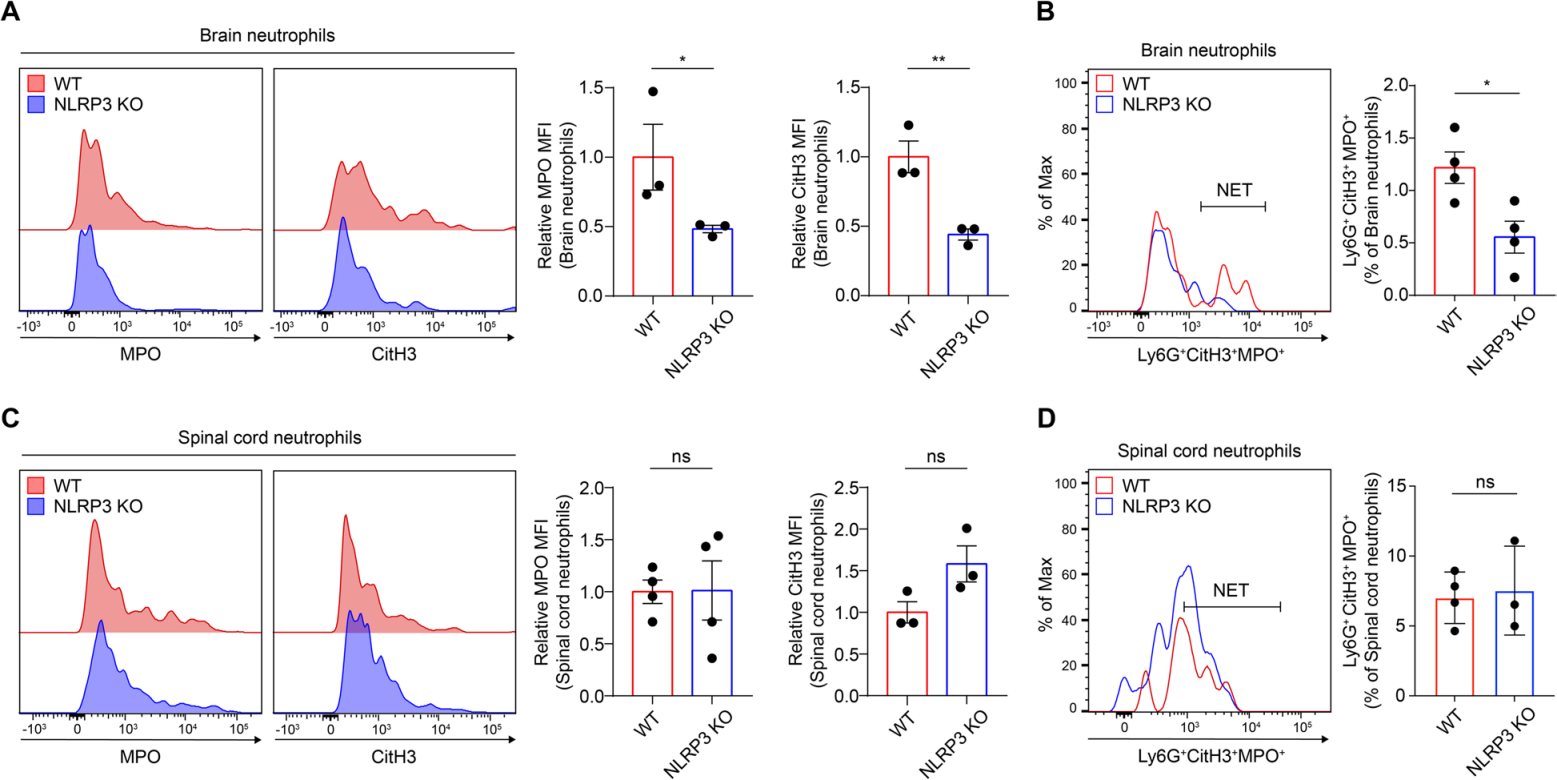
NLRP3 enhances NET formation predominantly in the brain of EAE pathogenesis. **A**–**D** Comparison of relative expression of NET components and percentage of NET formation in the brain and spinal cord of WT and NLRP3 KO mice at the disease peak. Representative histogram and relative MFI of extracellular MPO or CitH3, gated on CD11b^+^ and Ly6G^+^ cells, in both groups at peak disease on the (**A**) brain-infiltrated and (**C**) spinal cord-infiltrated neutrophils. Comparison of NET formation (Ly6G^+^CitH3^+^MPO.^+^) percentage in the (**B**) brain and (**D**) spinal cord from both groups. ns, non-significant. Data are acquired more than triplicates reiteration and presented as mean ± SEM. *P < 0.05, **P < 0.01

Our subsequent investigation aimed to determine whether NLRP3 modulates neutrophil functionality, specifically NET formation, in the CNS during EAE progression. To discern the differences in NET formation between WT and NLRP3 KO mice at the peak of the disease, extracellular MPO and CitH3 expression in CNS-infiltrated neutrophils were compared [24, 31]. We found that NLRP3 KO mice exhibited markedly reduced expression of MPO and CitH3 in the brain compared to WT mice (Fig. 3A). Moreover, NETs in neutrophils, identified by the co-expression of MPO and CitH3 in the Ly6G-positive gate, were significantly diminished in the absence of NLRP3 (Fig. 3B). However, the effects of NLRP3 deficiency in the spinal cord were inconsistent with those observed in the brain. In the spinal cord, neither individual MPO or CitH3 expression nor the co-expressed NET population significantly differed between WT and NLRP3 KO mice (Fig. 3C and D). Given the marked decrease in NET formation observed in the brains of NLRP3 KO mice at the peak of the disease, our results suggest that NLRP3 deficiency predominantly restricts NET formation in the brain during EAE progression.

### NLRP3 augmented NET formation in the brain in EAE pathogenesis

**Fig. 4:**
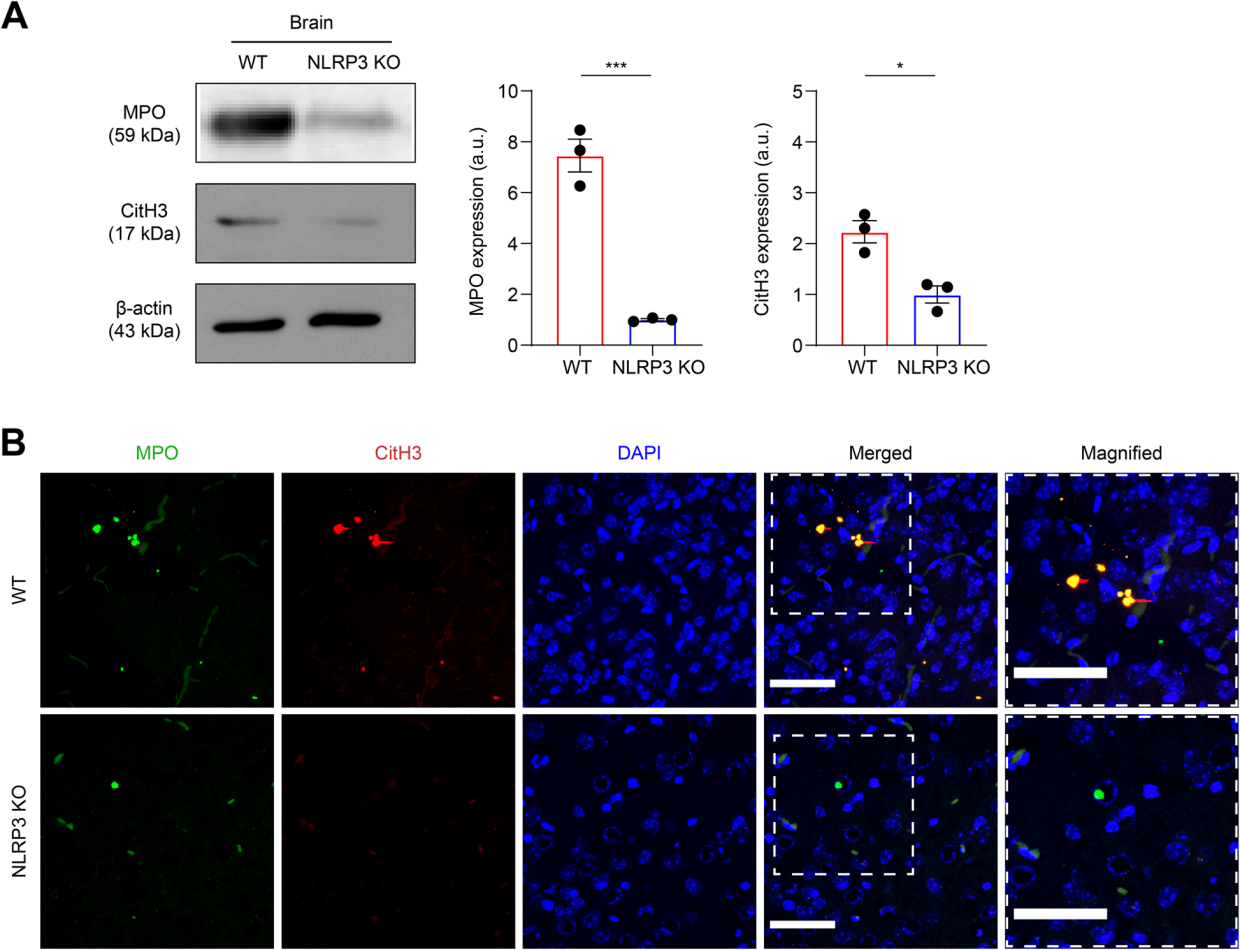
NLRP3 facilitates NET accumulation in the brain during EAE progression. **A** Immunoblotting and densitometric analysis for MPO, CitH3, and β-actin in the brain from WT and NLRP3 KO mice at peak disease. For quantification, the target expressions were normalized using β-actin. **B** Representative fluorescent images of NET formation in the brain from both groups, harvested at peak disease. Fluorescence images were acquired by staining for MPO (green), CitH3 (red), and nuclei (blue). Scale bar = 50 μm. Representative data from at least three independent experiments are shown. Data are driven from triple repeats and displayed as mean ± SEM. *P < 0.05, ***P < 0.001

To confirm NLRP3-mediated NET formation in the brain during EAE pathogenesis, the level of NET formation in EAE-induced brain tissue was quantified at the peak of the disease. Immunoblotting was performed to detect MPO and CitH3 expression in whole brain tissue lysate [12]. Consistent with our previous data, we confirmed that NLRP3 deficiency reduced NET protein accumulation in the brain (Fig. 4A). Supporting these observations, histological examination of EAE-induced brains revealed fewer NET-indicator-staining areas in NLRP3 KO mice than in WT mice (Fig. 4B) [24, 44]. Collectively, our results suggested that NLRP3 plays a deleterious role in the pathogenesis of EAE by promoting excessive NET formation in the brain.

### NLRP3 potentially enhanced NET formation by modulating CXCR2 and CXCR4 expression of neutrophils during EAE pathogenesis

**Fig. 5:**
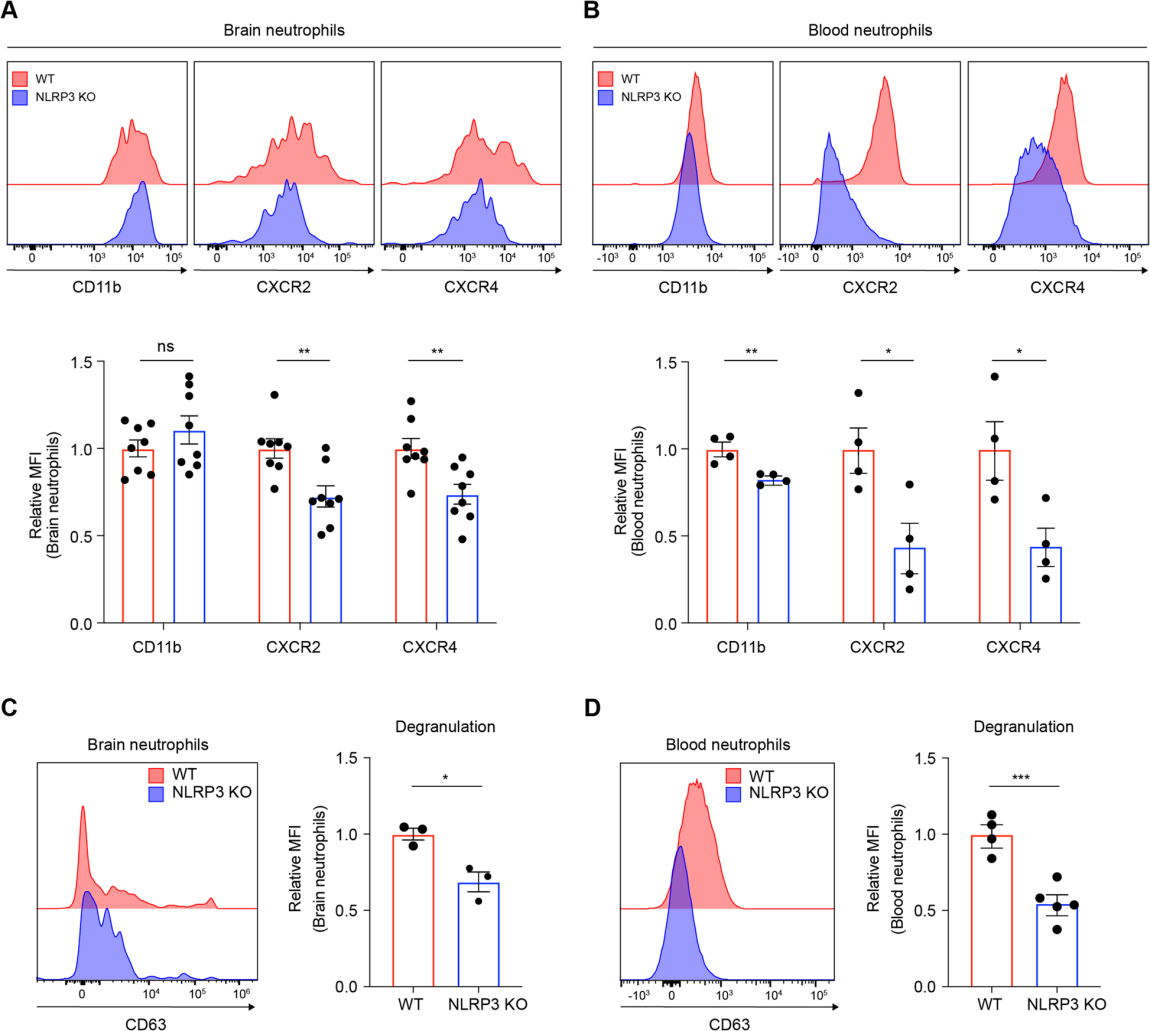
NLRP3 enhances CXCR2 and CXCR4 expression and degranulation in neutrophils, supporting NET formation during EAE pathogenesis. **A**–**D** NET-associated neutrophil phenotypical characteristics were compared between WT and NLRP3 KO mice. Analysis of the relative MFI of extracellular CD11b, CXCR2, and CXCR4 on neutrophils (gated on CD11b^+^ and Ly6G^+^ cells) from the (**A**) brain and (**B**) circulating neutrophils in both groups, as shown by representative histogram. Representative histogram and relative MFI of extracellular CD63 on neutrophils (gated on CD11b^+^ and Ly6G.^+^ cells) from both groups at the disease peak in the (**C**) brain and (**D**) circulating neutrophils. ns, non-significant. Data are obtained from at least three independent experiments and presented as mean ± SEM. *P < 0.05, **P < 0.01, ***P < 0.001

Given the observed reduction in NET formation at the peak of the disease in NLRP3-deficient mice, we aimed to further investigate whether NLRP3 converts the neutrophil phenotype, thereby enhancing NET formation during EAE pathogenesis [1, 45]. The association of CXCR2 with NET formation has been documented, as demonstrated by a significant reduction in NET formation in neutrophils from individuals with chronic obstructive pulmonary disease (COPD) following treatment with a selective CXCR2 antagonist [46]. The connection between CXCR4 and NET formation is corroborated by a study involving plasmodium-infected red blood cells. This investigation revealed that the release of macrophage migration inhibitory factor (MIF) from infected cells subsequently induced NET formation in neutrophils, and this mechanism was found to be dependent on CXCR4 [47]. Based on these previous reports suggesting that the chemokine receptors CXCR2 and CXCR4 are associated with NET formation [46–48], we explored whether NLRP3 has the capacity to modify the surface expression levels of these receptors in neutrophils during the peak of the disease. Our investigations focused on the properties of brain-infiltrating neutrophils, as we observed that NLRP3 predominantly inhibited NET formation in the brain. Brain-infiltrated neutrophils in NLRP3 KO mice showed decreased expression levels of CXCR2 and CXCR4, as compared to WT mice (Fig. 5A). Furthermore, we investigated their expression levels in circulating neutrophils, which also showed diminished expression levels of CXCR2, CXCR4, and CD11b under NLRP3 deficiency (Fig. 5B).

As a characteristic of hyperactivated neutrophils, degranulation is also a prerequisite for releasing primary granules, including the NET components NE and MPO [1, 33, 49]. A membrane protein associated with intracellular granules, CD63, is upregulated on the cell surface during the degranulation process, owing to the fusion of the granule and plasma membranes [33, 50]. In NLRP3 KO mice, neutrophil degranulation was also decreased in both blood and brain tissues, as compared to in WT mice (Fig. 5C and D). Altogether, these results are consistent with our previous observations and support our hypothesis that NLRP3 modulates neutrophils, predisposing them to form more NET during the pathogenesis of EAE.

### NLRP3 supported NET formation in ROS-dependent and PAD4-independent manner in the EAE-induced brain

**Fig. 6:**
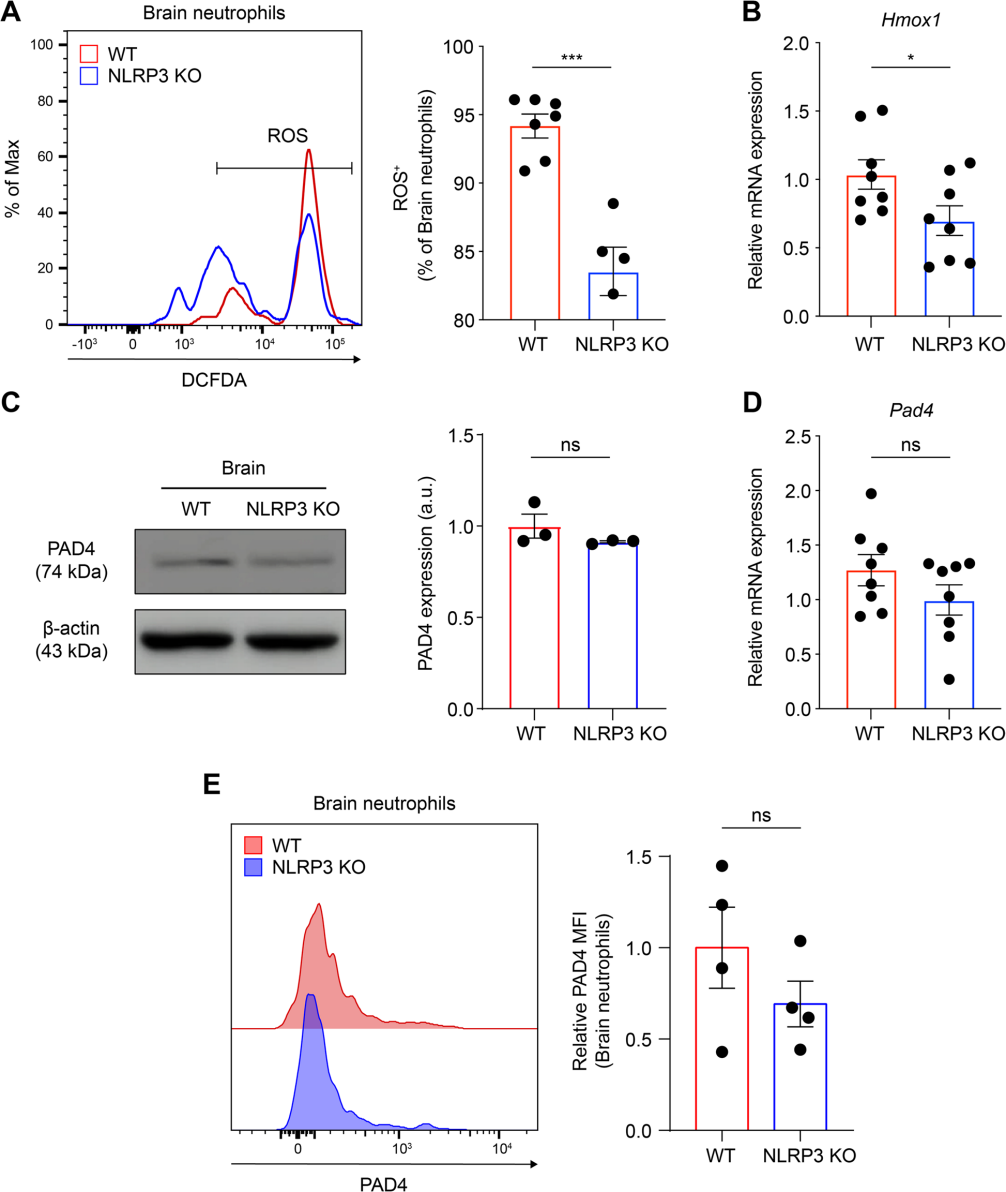
NLRP3 promotes ROS-dependent NET formation, regardless of PAD4, in EAE pathogenesis. **A** Representative scatter plots and percentages of ROS^+^ neutrophils (gated on CD11b^+^ and Ly6G^+^ cells) in the brain of EAE-induced WT and NLRP3 KO mice. **B** and **D** Total RNA was isolated from the whole brain of both groups at the disease peak, and (**B**) *Hmox1* and (**D**) *Pad4* expression were analyzed by quantitative real-time PCR (qRT-PCR). **C** Immunoblotting and densitometric analysis for PAD4 and β-actin in the brains collected from both groups at peak disease. For quantification, the target blots were normalized using β-actin. **E** Representative histogram and relative MFI of extracellular PAD4 on the brain-infiltrated neutrophils (gated on CD11b^+^ and Ly6G.^+^ cells) from both groups. ns, non-significant. Data are obtained from more than triplicated independent experiments and presented as mean ± SEM. *P < 0.05, ***P < 0.001

Next, we sought to gain insights into the molecular mechanisms underlying NET formation related to the neutrophil phenotype of increased chemotaxis in EAE pathogenesis. Initially, ROS production was measured, which is associated with primed neutrophils and NET formation [33, 51]. Classical NET formation depends on ROS levels; however, ROS-independent rapid NET formation has also been reported [52]. We investigated whether NLRP3 deficiency altered ROS production during autoreactive EAE pathogenesis. Using DCFDA, a fluorescent ROS indicator, and gating on Ly6G-positive neutrophils, ROS production was specifically measured in infiltrated brain neutrophils [32, 33]. Infiltrated neutrophils from NLRP3 KO mice exhibited decreased ROS production at EAE peak (Fig. 6A). To further determine oxidative stress in NLRP3 deficiency, we examined *Hmox1* gene expression instigated by ROS in whole brain tissue [33, 53]. Consistent with the reduced number of ROS^+^ neutrophils, *Hmox1* mRNA expression was lower in NLRP3 KO mice than in control mice at the peak of the disease (Fig. 6B). These results suggested that NLRP3 sustains ROS production during disease pathogenesis, thereby promoting NET formation. ROS activate PAD4, a crucial enzyme for NET formation, which further citrullinates histones and facilitates chromatin decondensation [54, 55]. Therefore, we explored whether NLRP3 deficiency also affects PAD4 expression in the brain tissue at the disease peak. No significant differences were observed in PAD4 expression at the protein and gene levels between the groups, despite changes in ROS (Fig. 6C-E). This discrepancy could be partly due to ROS itself, which also permits the translocation of NE, MPO, and other related enzymes [56, 57]. This translocation further promotes NET formation, which leads to the disruption of plasma membrane integrity. Accordingly, our findings suggest that NLRP3 facilitates NET formation primarily in an ROS-dependent manner, while operating independently of PAD4.

### NETs exacerbated EAE severity by augmenting Th1 and Th17 cell recruitment during EAE pathogenesis

**Fig. 7:**
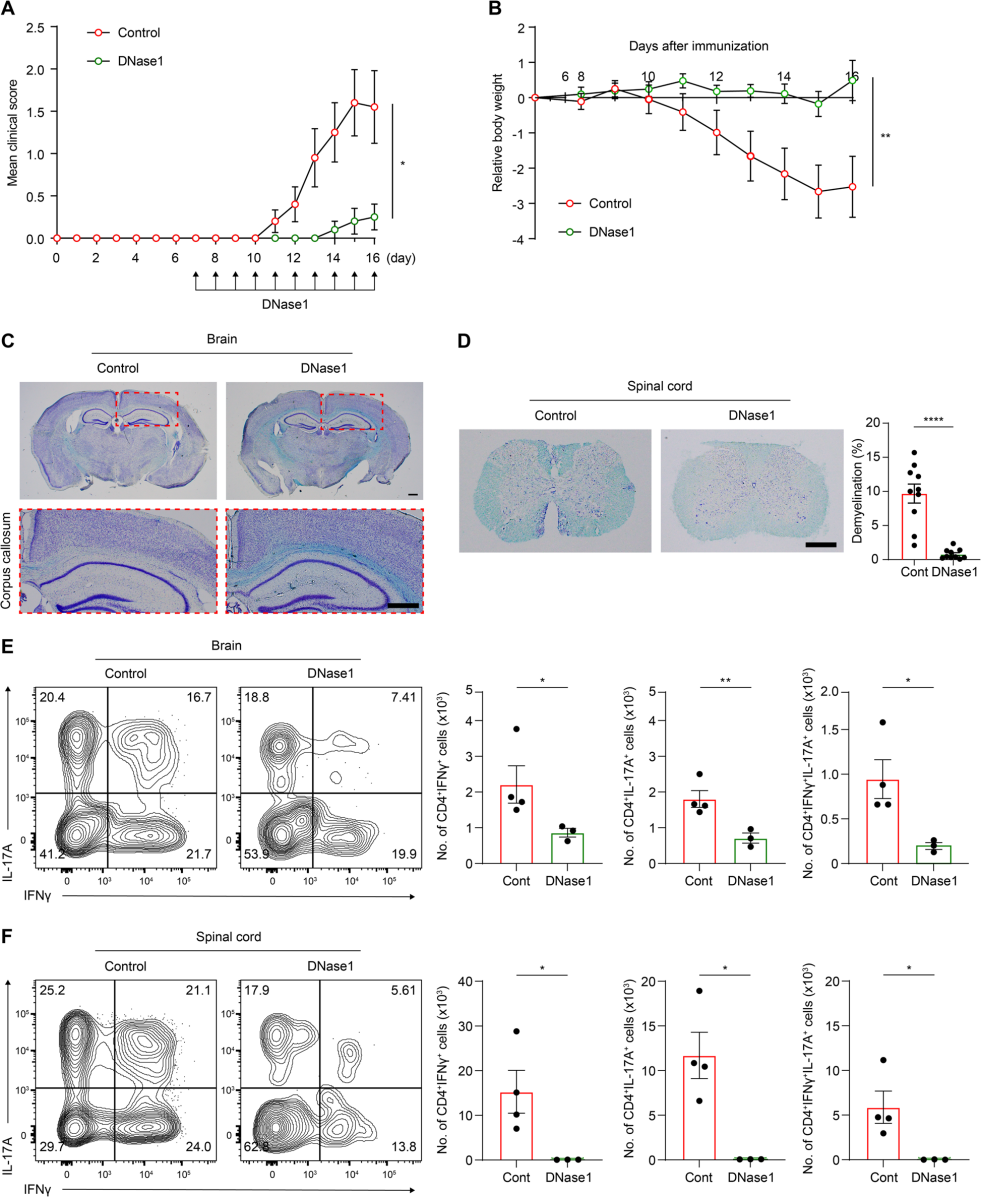
NET elimination alleviates EAE severity through reduced recruitment of Th1 and Th17 cells. **A**–**E** EAE severity and T cell recruitments were evaluated in control and DNase-1 groups. The DNase-1 group received DNase-1 from post- 7 days induction to peak of the disease, whereas the control was inoculated with the vehicle. **A** Clinical scores comparison between the control and DNase-1 groups. **B** Tracking of changes in body weight, following the administration in both groups. Both groups (**A** and **B**) were comprised of 10 mice each. **C** And **D** A comparison of demyelination degree in both groups, stained with luxol fast blue. **C** Upper, representative images of brain sections. Lower, the demyelinated area in the corpus callosum of the brain was presented. Scale bar = 500 μm. **D** Left, representative images of spinal cord sections. Scale bar = 500 μm. Right, the analysis of demyelination degree in both groups through thoracic to lumbar part of spinal cord. **E** and **F** Analysis of T cell populations, particularly CD4^+^IFNγ^+^, CD4^+^IL-17A^+^, and CD4^+^IFNγ^+^IL-17A.^+^ cell population, through flow cytometry in the (**E**) brain and (**F**) spinal cord collected from both groups. Data are obtained from at least three independent experiments and presented as mean ± SEM. *P < 0.05, **P < 0.01, ****P < 0.0001

Finally, we tested our hypothesis that NLRP3-supported NET formation could intensify EAE severity. Therefore, we assessed the potential deleterious effects of NETs using DNase-1 to dissolve the accumulated NETs during disease progression. DNase-1 was administered 7 days post-EAE induction at 24 h intervals [35, 36]. To evaluate disease severity following NET removal, we assigned disease scores and monitored the body weights of the mice following administration. The peak of disease was determined by the control group. The DNase-1-treated group achieved a lower disease score and experienced lesser weight loss than the control group (Fig. 7A and B). Similarly, we harvested brain and spinal cords at the disease peak and stained with the Luxol fast blue kit to gauge the extent of demyelination, as performed in Fig. 2C and D. Congruous with the scoring and weight monitoring results, elimination of NETs mitigated demyelination at disease peak (Fig. 7C and D). Hence, our data suggested that the removal of NETs could attenuate the severity of EAE. On top of examining symptom severity and demyelination degree, We further examined the effect of NET elimination on Th1 and Th17 cell recruitment, which are major contributors to demyelination in this disease [6]. A reduction in the population of CD4^+^IFN-γ^+^ (Th1 cells), CD4^+^IL-17A^+^ (Th17 cells), and CD4^+^IFN-γ^+^IL-17A^+^ was noted upon DNase-1 administration in the EAE-induced brain and spinal cord (Fig. 7E and F). In summary, these findings suggested that NLRP3-facilitated NET formation exacerbated the demyelination and severity of EAE by promoting the recruitment of Th1 and Th17 cells to the CNS during EAE progression. This finding reinforces our understanding of the deleterious role of NLRP3-supported NET formation in EAE pathogenesis and offers potential avenues for therapeutic interventions.

## Discussion

MS is a prevalent autoimmune disease that results in severe neurological dysfunction. However, a definite cure remains elusive owing to its complex pathology [1–3]. Our research demonstrates that NLRP3 enhances the propensity of neutrophils to generate ROS-dependent NETs by upregulating the expression of chemokine receptors in the progress of EAE. This NLRP3-facilitated NET formation appears to exacerbate the disease, potentially by stimulating the recruitment of Th1 and Th17 cells. Consequently, this study proposes a novel therapeutic approach for MS, with a primary emphasis on mitigating the recruitment of Th cells through the inhibition of NLRP3-supported NET formation.

Although previous studies have shown that released DNA induces inflammasome activation and, for example, AIM2 is involved in neutrophil DNA-mediated inflammasome activation [58], our findings indicate the probable existence of a positive feedback loop between NLRP3 and NET formation in neutrophils [24, 59, 60]. Owing to its role in signaling the innate immune response, NLRP3 could be activated during EAE progression, thereby enhancing NET formation in neutrophils. These NET may further stimulate NLRP3 activation. We propose that this cycle can be amplified through the enhanced expression of NET-associated chemokine receptors CXCR2 and CXCR4 by NLPR3. CXCR2 is a well-known cytokine receptor that guides neutrophils to inflamed sites [61]. Notably, we did not observe a corresponding increase in neutrophil infiltration following the upregulation of these receptors by NLRP3. Therefore, the mechanisms by which NLRP3 enhances CXCR2 and CXCR4 expression, and their subsequent effects, remain unclear. In addition, regarding the observation that NLRP3 deficiency exclusively influences CD11b expression in circulating neutrophils, it is important to note that CD11b is a chemokine receptor closely associated with cell adhesion and migration [62, 63]. Based on this observation, we inferred that increased expression of CD11b, which is likely required for these processes, becomes redundant in the context of brain-infiltrated neutrophils. This finding may imply a differential role of CD11b in neutrophils depending on their location and stage of migration, a concept expressed in previous studies [62–65].

Moreover, as we established the pivotal role of NLRP3 in NET formation in brain-infiltrated neutrophils, we primarily focused on the properties of these neutrophils. However, why NLRP3 has a minor effect on the spinal cord will be the next research goal. This discrepancy in location-specific characteristics of neutrophils is supported by a previous study suggesting that neutrophils significantly contribute to brain but not to spinal cord damage in EAE pathogenesis, as evidenced by neutrophil depletion [66]. Furthermore, it was noted that alterations in the circulating expression of CXCR2 and CXCR4 exhibited no discernible impact on the spinal cord. Drawing from existing references elucidating the nuanced pathogenesis of EAE in distinct anatomical regions, such as the brain and spinal cord, we speculate that disparate microenvironmental factors in these regions may contribute to the observed variations in NET formation, sustained by NLRP3 [67]. We recognize that this hypothesis necessitates further exploration to elucidate the precise mechanisms underpinning the observed phenomenon.

Given that NLRP3 deficiency reduces NET formation in the brain at disease peak, we initially investigated whether NLRP3 was associated with NET formation or its subsequent elimination. Although a slight upregulation was noted in DNase-1 levels in the brain of NLRP3-deficient group without statistical significance, there was insufficient evidence to hypothesize that NLRP3 maintains the homeostasis of DNase-1 expression thereby the NLRP3 deficiency induced less NET accumulation during EAE pathogenesis. Thus, our research hypothesis leaned towards the pro-inflammatory role of neutrophil NLRP3 in enhancing NET formation.

Remarkably, we also observed that NET removal via DNase-1 not only diminished the recruitment of conventional CD4^+^IFN-γ^+^ (Th1) and CD4^+^IL-17A^+^ (Th17) cells, but also reduced the presence of the CD4^+^IFN-γ^+^IL-17A^+^ population in the CNS at disease peak. We hypothesized that this population may be significantly influenced by NETs and play a key role in disease alleviation. A previous study suggested that in vitro-differentiated Th17 cells can develop the capacity to produce both IL-17A and IFN-γ in EAE, which distinguished as ex-Th17, displayed attributes more similar to Th1 cells, rather than Th17 cells within EAE lesions in vivo [68, 69]. These observations imply that NETs play a significant role in shaping the phenotype and effector functions of T cells in EAE pathogenesis. The specific mechanisms by which NETs modulate Th cell phenotypes, including their potential influence on the intrinsic programming of effector CD4^+^ T cell subsets and the key parameters affecting T cell effector activity during EAE progression, require further elucidation. There are three potential underlying mechanisms involved in the interaction between NETs and T cells during the pathogenesis of EAE. Firstly, NETs themselves can directly stimulate CD4^+^ T cells by reducing their activation threshold, leading to T cell priming [70]. Secondly, as previously reported, NETs and their associated histone complexes may induce the differentiation of Th17 cells [70, 71]. Lastly, once NETs are released, antigen-presenting cells (APCs) recognize, process, and present them as self-antigens to T cells. Furthermore, the IL-17 cytokine as well as GM-CSF produced from activated T cells, can influence further neutrophil recruitment and stimulation, creating a pro-inflammatory feedback loop [72, 73]. Therefore, the mechanisms underlying the niche formation and interaction between NETs and T cells in the brains of MS patients remain enigmatic.

Although our results suggest that the elimination of NET via NLRP3 inhibition could alleviate the disease, these results may not fully translate in actual patients with MS, owing to the inherent limitations of the EAE model. Although EAE is a well-established animal model for bench research on MS, this model has limitations in mimicking the detailed pathology of MS [6, 7]. For instance, the MOG_35-55_-induced EAE model fails to stimulate B cells, which are also notable contributors to disease progression. Certain researchers have applied an adaptive T cell-transfer EAE-inducing model to overcome this limitation and more accurately mimic EAE pathology [74]. However, we used the MOG_35-55_-induced EAE model because our primary focus was on neutrophils and their extracellular traps. Moreover, our results provide evidence supporting the potential application of NLRP3 inhibitors to alleviate disease severity in patients with MS. However, it is crucial to consider contradictory reports on the anti-inflammatory role of NLRP3 in certain diseases. For instance, NLRP3 deficiency can exacerbate inflammation during herpes simplex virus type 1 ocular infection [75].

Furthermore, after focusing on the role of NLRP3 in NET formation, we exploited DNase-1 and systemically inoculated it to verify the deleterious effects of NETs sustained by NLRP3. Although our findings suggest that NETs influence disease progression as well as Th1 and Th17 cell recruitment, it is important to note that the technique used in this study to eliminate NETs has limitations in ascertaining the role of NET formation, particularly in the brain, as it abolished NETs not only in the brain but also systemically. This might cause the heightened potency of DNase-1 while eliminating all types of NETs induced by various signaling pathways. Therefore, the specific significance of increased NETs in the brain, driven by NLRP3, in disease progression remains unclear. Given the potential perturbation of NLRP3 inhibition, limitations of the EAE model for MS, and broad effects of DNase-1 administration, a comprehensive investigation is required to consider the possible discrepancies and side effects before clinical application.

In summary, our findings indicate that NLPR3 enhances ROS-dependent NET formation in the diseased brain by modulating neutrophil phenotypes, leading to Th1 and Th17 cell recruitment, and worsening disease progression. Our study holds significant value as it reveals the connection between NLRP3 and NET formation, particularly in EAE pathology.

## Conclusion

Herein, we demonstrated neutrophil infiltration and substantial NET formation in the CNS during EAE pathogenesis. According to our results, NLRP3 deficiency notably reduced NET accumulation, predominantly in the brain, at disease peak. Further investigation of this NLRP3-supported mechanism of NET formation revealed that NLRP3 may make neutrophils more prone to form ROS-dependent NET by modifying the expression of CXCR2 and CXCR4 in brain-infiltrated neutrophils. Thus, we verified that NET is an etiological factor in EAE pathogenesis through NET elimination, attenuating demyelination and the severity of the disease by decreasing Th1 and Th17 cell recruitment. Collectively, our study holds significance by suggesting a new therapeutic approach to reduce T cell recruitment and alleviate the progression of MS through the inhibition of NLRP3-supported NET formation.

### Supplementary Information

**Additional file 1: Supplementary Fig. 1.** Gating strategy for Ly6G^+^CitH3^+^MPO^+^ population via flow cytometry analysis. Initially, whole cells were chosen based on a forward scatter area vs. side scatter area dot plot, and singlets were further refined in a forward scatter width vs. forward scatter height dot plot and side scatter width vs. side scatter height dot plot. To distinguish live cells, Zombie Aqua dye, exclusively binding to dead cells, was employed, and the negative cells were gated. Subsequently, neutrophils were identified by gating for Ly6G^+^ expression in a dot plot. Within the neutrophil gate, the CitH3^+^ population was selected. Finally, within the CitH3^+^ population gate, the MPO^+^ population was selected, representing the co-localized population of Ly6G, CitH3, and MPO.

## Data Availability

Data and materials are available by contacting the corresponding author.

## Abbreviations

CFA: Complete Freund’s adjuvant
CitH3: Citrullinated histone H3
CNS: Central nervous system
DAMPs: Damage-associated molecular patterns
EAE: Experimental autoimmune encephalomyelitis
HBSS: Hanks’ balanced salt solution
LPS: Lipopolysaccharide
MS: Multiple sclerosis
MPO: Myeloperoxidase
NE: Neutrophil elastase
NET: Neutrophil extracellular trap
NLRP3: NLR family pyrin domain containing 3
OCT: Optimal cutting temperature
PAD4: Protein arginine deiminase 4
PAMPs: Pathogen-associated molecular patterns
PBS: Phosphate-buffered saline
PTX: Pertussis toxin
ROS: Reactive oxygen species
SIP: Stock isotonic Percoll
