# Supplementary figures and images for: NLRP3 exacerbates EAE severity through ROS-dependent NET formation in the mouse brain
Source: Cell Commun Signal. 2024 Feb 2;22:96. doi: 10.1186/s12964-023-01447-z (PMC10835891; doi:10.1186/s12964-023-01447-z)

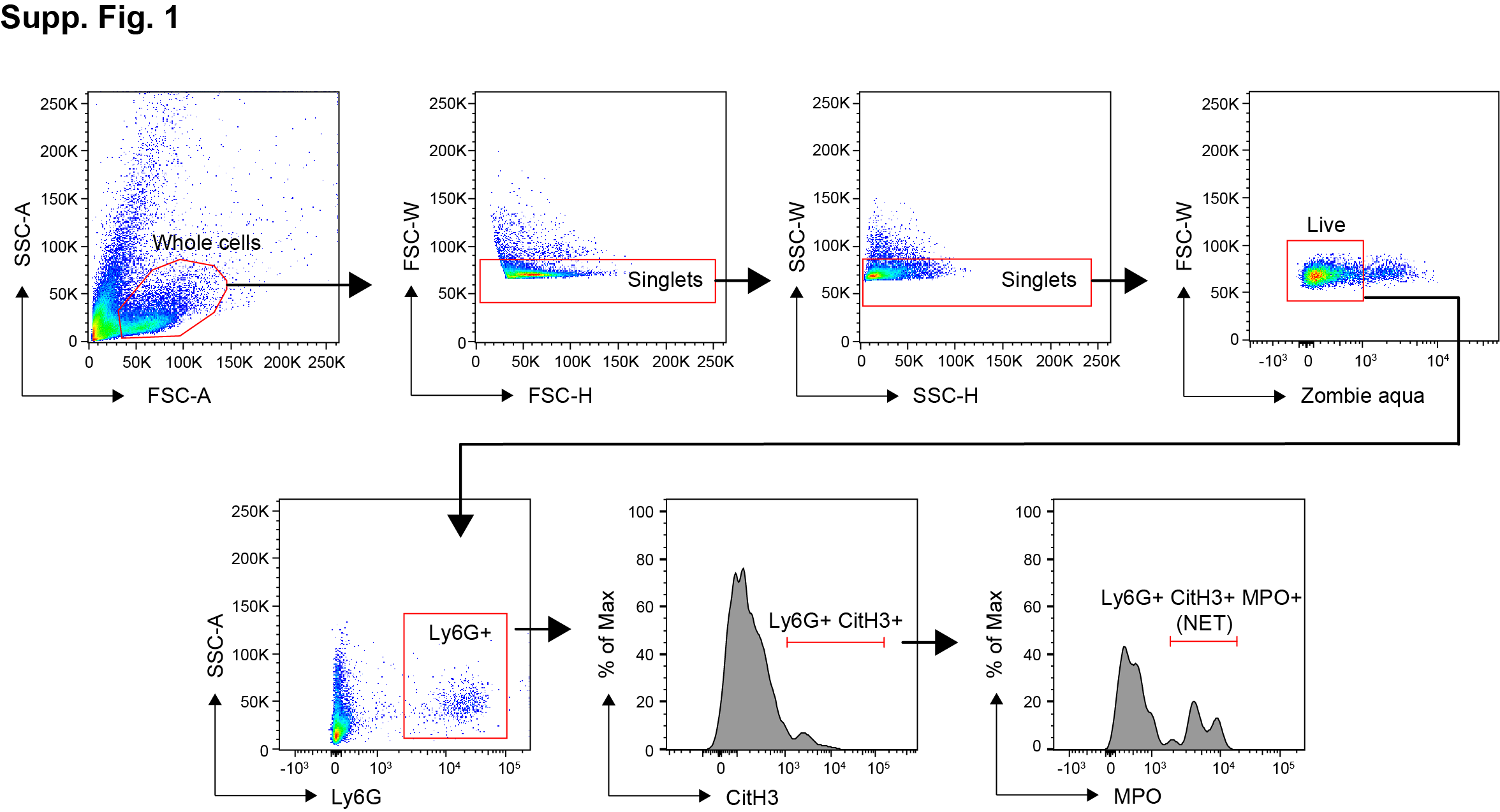

Supplement: Supplementary file 1 — Additional file 1: Supplementary Fig. 1. Gating strategy for Ly6G+CitH3+MPO+ population via flow cytometry analysis. Initially, whole cells were chosen based on a forward scatter area vs. side scatter area dot plot, and singlets were further refined in a forward scatter width vs. forward scatter height dot plot and side scatter width vs. side scatter height dot plot. To distinguish live cells, Zombie Aqua dye, exclusively binding to dead cells, was employed, and the negative cells were gated. Subsequently, neutrophils were identified by gating for Ly6G+ expression in a dot plot. Within the neutrophil gate, the CitH3+ population was selected. Finally, within the CitH3+ population gate, the MPO+ population was selected, representing the co-localized population of Ly6G, CitH3, and MPO. [file 12964_2023_1447_MOESM1_ESM.png]
